# Metformin: Metabolic Rewiring Faces Tumor Heterogeneity

**DOI:** 10.3390/cells9112439

**Published:** 2020-11-09

**Authors:** Mario Cioce, Claudio Pulito, Sabrina Strano, Giovanni Blandino, Vito Michele Fazio

**Affiliations:** 1 Department of Medicine, R.U. in Molecular Medicine and Biotechnology, University Campus Bio-Medico of Rome, 00128 Rome, Italy; fazio@unicampus.it; 2 Oncogenomic and Epigenetic Unit, IRCCS Regina Elena National Cancer Institute, 00144 Rome, Italy; claudio.pulito@ifo.gov.it (C.P.); giovanni.blandino@ifo.gov.it (G.B.); 3 SAFU Unit, Department of Research, Diagnosis and Innovative Technologies, IRCCS Regina Elena National Cancer Institute, 00144 Rome, Italy; sabrina.strano@ifo.gov.it; 4 Institute of Translation Pharmacology, National Research Council of Italy (CNR), 00133 Rome, Italy

**Keywords:** metformin, cancer stem cells, autophagy, metabolic heterogeneity, mitochondria, OXPHOS, therapeutic target, ETC, STAT3, NFkB

## Abstract

Tumor heterogeneity impinges on all the aspects of tumor history, from onset to metastasis and relapse. It is growingly recognized as a propelling force for tumor adaptation to environmental and micro-environmental cues. Metabolic heterogeneity perfectly falls into this process. It strongly contributes to the metabolic plasticity which characterizes cancer cell subpopulations—capable of adaptive switching under stress conditions, between aerobic glycolysis and oxidative phosphorylation—in both a convergent and divergent modality. The mitochondria appear at center-stage in this adaptive process and thus, targeting mitochondria in cancer may prove of therapeutic value. Metformin is the oldest and most used anti-diabetic medication and its relationship with cancer has witnessed rises and falls in the last 30 years. We believe it is useful to revisit the main mechanisms of action of metformin in light of the emerging views on tumor heterogeneity. We first analyze the most consolidated view of its mitochondrial mechanism of action and then we frame the latter in the context of tumor adaptive strategies, cancer stem cell selection, metabolic zonation of tumors and the tumor microenvironment. This may provide a more critical point of view and, to some extent, may help to shed light on some of the controversial evidence for metformin’s anticancer action.

## 1. A Brief Intro to Tumor Heterogeneity

“Tumor societies are highly adapted for survival” is a definition from Dr. Gloria Heppner which captured, 36 years ago, the deep meaning of tumor heterogeneity [1]. In fact, it is now recognized that tumors at diagnosis are a kaleidoscopic collection of genomically, epigenetically, metabolically, and topographically different subclones, arranged into a complex ecosystem. The coexistence of different cell subpopulations within a single tumor plays crucial roles during all stages of carcinogenesis and poses challenges for clinical treatments by directly impinging on adaptive stress tolerance [2,3,4,5]. Genetic and epigenetic events may draw the landscape for tumor heterogeneity, which is then greatly fueled by dramatic remodeling of the secretome, paracrine signaling and adaptive metabolism [2,6].

Therapy-induced stress may further promote metabolic heterogeneity, favoring dynamic shifts between aerobic glycolysis and mitochondrial oxidative phosphorylation (OXPHOS) [7,8,9]. In more detail: advanced modelling inferred from correlating gene expression and metabolic foot-printing of tumors, has shown that cancer cells are capable of adopting metabolic intermediate states in a very dynamic way and to a larger extent than normal cells [10]. Thus, tumors may use both aerobic glycolysis and OXPHOS, to a variable extent, depending on tumor stage and time [11,12,13,14]. The AMP-activated protein kinase (AMPK) is the main molecular rheostat governing such plasticity [15]. Thus, targeting metabolic adaptation to subvert therapeutic resistance is a clinically viable approach [14,16]. In this review we will discuss metformin and its mechanism of action (MoA). We discuss how such a long-lived drug, may impinge on adaptive strategies of tumors, by targeting both converging mechanisms (common to both normal and transformed cells) and context-specific alterations.

## 2. Metformin Elicited Signaling Disturbance 

Metformin (N′,N′-dimethylbiguanide) represents the most frequently administered drug to treat patients with metabolic syndrome and type 2 diabetes, worldwide. Its use has spanned over 60 years and is partially due to a very positive risk–benefit profile [17,18,19,20]. Systemically, metformin therapy lowers blood glucose in type 2 diabetes patients by targeting hepatic gluconeogenesis and by increasing glucose uptake in the peripheral tissues, mainly muscles, and indirectly reduces the insulin blood levels by counteracting insulin resistance [19,21]. Before digging into the relationships between metformin and cancer, we believe it may be useful to quickly review the main mechanism of action (MoA) of metformin in diabetic, non-cancer patients.

**Figure 1:**
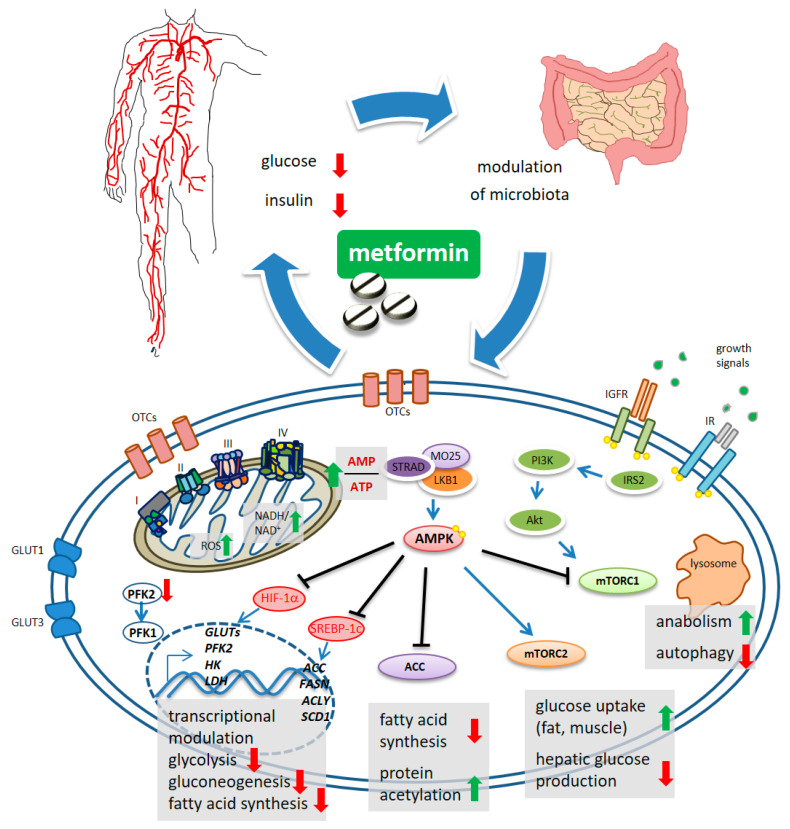
Schematic model of metformin action. Systemically, metformin lowers the glucose levels and, indirectly, reduces insulin levels. Metformin crosses the plasma membrane at least partially through organic cation transporters (OCTs and related) and enters mitochondria where it affects complex 1 coupling and causes altered redox status and increased AMP/ATP ratio. This latter activates, allosterically, the kinase LKB1 which phosphorylates AMPK. mTORC1 functions as an environmental sensor and is activated by insulin signaling and growth factors to promote anabolic metabolism and to inhibit autophagy. Metformin-stimulated AMPK reverses mTORC1 actions. mTORC2 is activated by AMPK and stimulates increased glucose entry into muscles and reduced glucose production in the liver. ACC inhibition reduces fatty acid synthesis and may increase collective protein acetylation (increased acetyl-CoA) thus exerting transcriptional modulation. Metformin-stimulated-AMPK controls transcriptionally hepatic gluconeogenesis. Metformin interferes with the increase in HIF-1α and the control of glycolytic genes and GLUT transporters, including the expression of PFK2 which increases the levels of the metabolite fructose-2,6-P_2_, allosterically activating PFK-1. Downregulated expression of ACC, FASN, ACLY and SCD1 was promoted by metformin, through interference with SREBP-1c and TR4.

Undisputedly, the finding that metformin indirectly activates the adenosine 5′-monophosphate protein kinase (AMPK) [22,23,24], represented an important turn in the still unfinished quest for its mechanism of action (MoA) [25] (Figure 1). The “secret recipe” in metformin’s MoA consists of its hydrophilic nature, cationic behavior, Fe and Cu-binding properties and a pK_a_ within the physiological pH range [26]. Metformin accumulates in the mitochondria of intact cells by virtue of its positive charge [27] (Figure 1), causing inhibition of complex 1 of the respiratory chain [28]. In detail, metformin interferes with the coupling of redox and proton transfer domains in complex 1, resulting in altered redox status at the mitochondria and cytosol [26,29] and reactive oxygen species (ROS) accumulation [27,30]. The reduced phosphorylation of adenine nucleotides and accumulation of AMP allosterically determines the liver kinase B1-STE20-related pseudokinase-calcium binding protein-39 (LKB1-STRAD-CAB39)-mediated activation of AMPK [31]. Furthermore, “non-canonical” activation of AMPK takes place at lysosomes and is triggered either by changes in fructose-1,6-bisphosphate (F1, 6P2) which affects LKB1-mediated phosphorylation [32], or by galectin-9-promoted transforming growth factor-β-activated kinase-1 (TAK1)-mediated phosphorylation [33], the latter being linked to induction of autophagy [34,35]. Interestingly, activation of AMPK seems to be spatially and temporally regulated: a mild increase in AMP may activate the cytoplasmic and lysosomal pool while a more sustained increase in AMP may promote phosphorylation of AMPK by the LKB1 complex in mitochondria [36]. This may have a functional consequence in light of the topographic constraints and gradients existing within both normal tissues and in the tumor mass.

Regarding the inhibition of gluconeogenesis, recent evidence shows that the early, acute downregulation of gluconeogenesis is AMPK independent and is possibly linked to compromised functioning of complex 1 that causes unbalanced NADH/NAD in mitochondria and an altered cytosolic redox state [26,37] and/or by inhibition of the mitochondrial glycerol-phosphate dehydrogenase (mGPD) [38].

The late inhibition of gluconeogenesis happens transcriptionally and is AMPK dependent. In this latter process, the small heterodimer partner (SHP), a transcriptional co-repressor, and phosphorylation of CREB binding protein (CBP) by AMPK-PKCι/λ (protein kinase C) were shown to play critical roles [39,40,41,42] (Figure 1). Additionally, even with some context specificity, AMPK targets additional metabolic enzymes such as HK2, glycogen synthase (GS), and hydroxy-methyl-glutaryl-CoA reductase (HMGCR) [43,44]. Metformin also downregulated the expression of glucose transporters (GLUT1, GLUT3) and of glycolytic enzymes such as hexokinase 2 (HK2), 6-phosphofructo-2-kinase/fructose-2,6-biphosphatase 4 (PFKFB4), pyruvate kinase (PKM) and lactate dehydrogenase A(LDH) [45]. In hepatocarcinoma cells (HCC), metformin inhibited phosphofructokinase-1 (PFK1) activity by suppressing the expression of PFK2, thereby reducing the allosteric activation of PFK1 by fructose-2,6-bisphosphate [46] (Figure 1). These effects were achieved through metformin-induced inhibition of HIF-1α activity and its binding to the hypoxia-responsive elements (HRE) within the promoter region of these genes [47]. Collectively, the effect of metformin-activated AMPK accounts for increased catabolism and decreased anabolism by modulating protein synthesis, lipid homeostasis, glycolysis—and mitochondrial homeostasis, in addition to transcriptionally modulating gluconeogenesis (Figure 1).

### 2.1. Metformin Modulates the Activity of mTORC1 and mTORC2 Complexes

Mammalian target of rapamycin (mTOR) is the core of two, functionally distinct, multiprotein complexes, mTOR complex 1 (mTORC1) and mTORC2 [48], oppositely modulated by metformin via AMPK [49,50]. mTORC1 activation exerts anabolic effects (through increased ribosome biogenesis, lipid, nucleotide and protein synthesis) and suppresses autophagy. The mTORC1 is activated by insulin and growth factors via phosphatidyl-inositol 3-kinase (PI3K)/AKT [50]. Activated AMPK directly phosphorylates tuberous sclerosis complex 2 (TSC2), thereby inhibiting mTORC1 [49]. mTORC1 signaling can also be inhibited by a metformin-sensitive Ras-related GTPase, as shown in mouse embryo fibroblasts [51]. On the other hand, metformin-stimulated AMPK activates mTORC2, thereby promoting cell survival and systemically reducing hepatic glucose production [52].

### 2.2. Metformin Inhibits Fatty Acid Synthesis

Another direct AMPK target protein highly relevant for cellular energy consumption, is the acetyl-CoA carboxylase (ACC), deactivated by AMPK via phosphorylation [53]. The inhibition of ACC activity decreased fatty acid synthesis consequent to a reduced conversion of acetyl-CoA to malonyl-CoA [54] (Figure 1). ACC inhibition increases collective protein acetylation and thus, may exert transcriptional modulation [43,44] (Figure 1). Additionally, phosphorylation by metformin-activated AMPK inhibited the proteasome-mediated degradation of insulin-induced gene 1 (Insig-1), which in turn reduced the activating cleavage of the transcription factor sterol regulatory element-binding protein-1c (SREBP-1c) and consequently, reduced lipogenic gene expression [55] (Figure 1). In a mouse hepatoma model, metformin decreased de novo fatty acid synthesis by reducing, transcriptionally, the expression of acetyl CoA carboxylase, fatty acid synthase (FASN) and ATP citrate lyase (ACLY) [56]. Additionally, metformin impaired—AMPK-dependently—the binding and transactivation of the nuclear receptor TR4 to its responsive elements in the stearoyl-CoA desaturase-1 (*SCD1)* promoter, in hepatocytes [57] (Figure 1). 

### 2.3. Metformin Modulates Gut Microbiota

Remodeling the gut microbiota mediates the therapeutic effects of metformin and is responsible for its known gastrointestinal side effects [58,59]. In fact, there is long-known evidence showing that the full glucose-lowering effect of metformin is bound to its oral administration [60] and that antibiotics may blunt the effect of metformin in animal models [61]. Additionally, the concentration of metformin in the jejunum was estimated to be from ten to a few hundred times higher than in plasma [62]. The changes in microbiota elicited by metformin in diabetic patients can be relevant for its anticancer action, given the involvement of gut microbiota in the pathogenesis of colorectal cancer (CRC) and other solid tumors [63]. For instance, an effect of metformin on the abundance of intestinal *Akkermansia muciniphila* has been reproducibly reported [61,64]. *A. Muciniphila* was shown to increase the abundance of gut-targeted CD4+ T cells, providing an adjuvant effect to the action of anti- programmed cell death -1 (PD-1) agents, in animal models of melanoma and non-small cell lung cancer (NSCLC) [65] and in a model of microsatellite-stable (MSS) colorectal cancer [66].

## 3. Metformin in Cancer Patients

Epidemiological studies suggest preventive effects of metformin on many types of human cancers [67]. A large, population-based, case–control study in the Tayside region of Scotland evidenced that in type 2 diabetic patients, using metformin was associated with reduced risk of breast cancer [68]. This was further supported by evidence in liver, colon, and pancreatic cancer patients, obtained elsewhere [69]. In therapeutic settings, improvement of overall survival (OS) was observed in breast, pancreatic, liver, colorectal and prostate cancer, generally in window-of-opportunity trials [70,71]. Effects of metformin were shown also in non-diabetic colorectal-cancer (CRC) and breast cancer patients [72,73]. More recently, an observational, large, population based study on 315,000 patients has partially challenged the above findings, except for prostate and pancreas cancers [74]. 

### 3.1. Metformin in Cancer Cells: Influence of Intra- and Inter-Tumor Heterogeneity 

**Figure 2:**
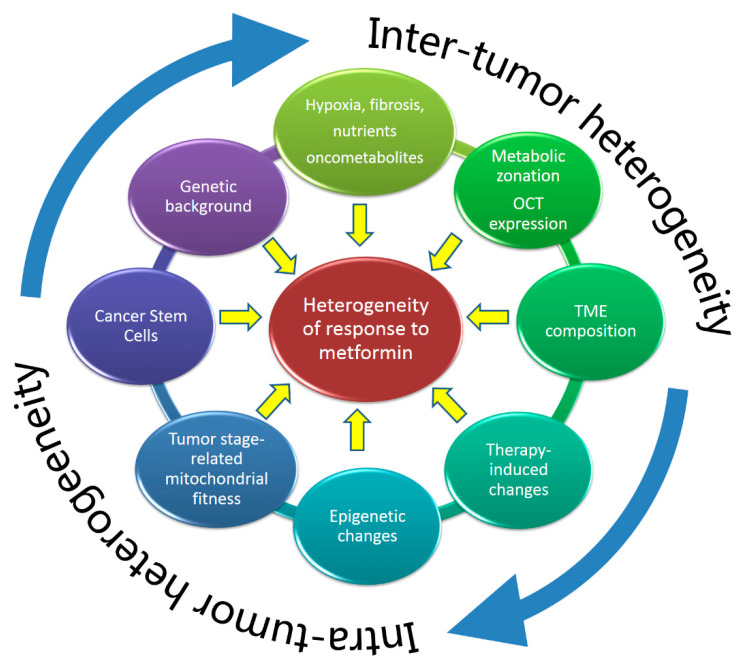
A schematic representation of the main factors modulating the anticancer effects of metformin. The genetic background of the tumor may primarily influence the inter-tumor heterogeneity. The extent of hypoxia and fibrosis and, ultimately, the nutrient availability and the release of oncometabolites may greatly vary among patients as a function of tumor size and location, thus creating spatially defined metabolic zonation phenomena inside the tumor. The composition of the tumor microenvironment (TME) influences and is influenced by therapy-induced changes within the tumor which may trigger epigenetic reprogramming. Altogether, these factors promote the emergence of stress-adapting cancer cell subpopulations, with features of cancer stem cells (CSCs). The tumor stage-dependent change in mitochondrial fitness may contribute to the selective pressure driving the oligo-clonal emergence of CSCs. Those cell subpopulations may ultimately drive tumor metastasis and relapse and may resist metformin treatment as a single agent by acquiring dynamic metabolic states. Please note that the circular arrows indicate that both inter- and intra-tumor heterogeneity are self-fueling processes which strongly influence each other.

The anticancer effect of metformin dates back pretty far in time. In the late seventies, metformin and phenformin had already been shown to suppress chemical carcinogenesis in rats and to foster immunity in breast cancer patients [75,76]. Since then, there have been more than 5000 publications and dozens of evoked MoAs. This is noteworthy, considering that high blood insulin levels represent an important prognostic factor for many solid cancers, partly because of the proliferative signals delivered by the insulin receptor and the insulin-like growth factor receptor (IGFR) [77,78]. The ability of metformin to indirectly lower insulin in the blood by attenuating insulin resistance [79] represents a first important anti-cancer property of the molecule (Figure 1). Now, it may help to further consider some aspects of the inter- and intra-cancer heterogeneity which may prompt for a more critical understanding of metformin action in cancer and may justify the heterogeneity of results reported in the literature. Firstly, in tumors, metabolic routing of survival strategies is very dynamic [4]; it is usual that, in a tumor tissue, a fraction of the cells are in an aerobic glycolytic state, while a significant fraction are utilizing, to a various degree of efficiency, both OXPHOS and aerobic glycolysis [10]. This may be influenced by nutrient availability, local hypoxia and effects of the oncometabolites [11] (Figure 2). This may create a zonation phenomenon, recently described for cancer tissues [80,81] (Figure 2). For example, in human glioblastomas, the proximity to the blood vessels determines “zonation” of both “transcriptomically” and “metabolomically” distinct cell subpopulations [80]. Pertinently, the expression of organic cation transporter 3 (OCT3), considered the main organic cation transporter responsible for metformin uptake into the cells [82], can be very heterogeneous in a single tumor [83]. Further on this topic, only a variable fraction of tumor cells are in an epithelial-to-mesenchymal (EMT) state or possess mesenchymal features in vivo and the EMT state was shown to modulate sensitivity of breast cancer cells to metformin [84]. It is therefore likely that only a fraction of cells in a given region of the tumor, at a given time in its history, may be sensitive to metformin action.

It may be interesting to consider, besides intra-tumor heterogeneity, how the inter-tumor differences may shape metformin’s response. For example, a recent algorithm-assisted proteomic characterization of ten hepatocellular carcinoma tissues as compared to “normal” peri-tumoral ones, revealed a huge heterogeneity affecting most of the pathways analyzed and could be exemplified by a high variability in lactate production and glycogen accumulation. Consequently, all the analyzed tumors behaved differently to metformin challenge [85]. In this case, a possible difference in the biological stage of the tumors could be called in question: it is indeed known how the mitochondrial (and OXPHOS) fitness increases with the tumor stage (Figure 2). In fact, mitochondrial biology may vary with tumor stage, with a general increase in mitochondrial fitness supporting mature stages of tumor formation and progression, including resistance to therapy and metastasis [13]. 

In support of the above considerations, a recent pharmacogenomics study on both primary cultures and cancer cell lines has revealed a significant cell context-specific variability of response to metformin. Cell survival analysis, transcriptome and whole exome sequencing analysis were performed on both primary dermal fibroblasts and induced-pluripotent-Stem cells (IPS), derived from NSCLC patients. Cells of identical genotypes from different tissues or in different stages of development exhibited differential sensitivity to metformin. In detail, skin fibroblasts, iPS- derived FOXA2+ or NKX2.1+ lung endoderm cells showed very different responses to metformin treatment, despite being derived from the same patient [86]. In the same study, the authors identified specific genomic signatures of response to metformin, associated with single nucleotide variations (SNVs) and “small indels”, differentially represented in metformin-sensitive and -resistant cells [86]. 

It is also relevant to consider the contribution of the tumor microenvironment [87] to metformin’s effect and vice versa: metformin, as somehow expected, acts on the tumor microenvironment, thereby perturbing the tumor stroma crosstalk, as very recently shown in endothelial cells and ovarian cancer-associated fibroblasts (CAFs) [88,89,90]. As it follows, the heterogeneity of the tumor microenvironment, which is highly regarded as a determinant of tumor progression and response to therapy, may influence metformin action (Figure 2) [90]. 

These considerations, collectively, suggest the usefulness of combinatorial usage of metformin to target insensitive cell subpopulations and to increase the therapeutic window of the combined agent. This is detailed in the following paragraphs.

### 3.2. Molecular Mechanisms for Anticancer Effects: It May All Start from Mitochondria

#### 3.2.1. Dose of Metformin in Cancer Studies, a Long Debate with a Recent Twist

Firstly, some consideration regarding the dose of metformin used for cancer studies. Metformin doses used in cancer studies often exceed the feasible therapeutic plasma levels (0.465–2.5 mg/L or 2.8–15 mM) in humans. In fact, clinical studies use metformin up to 2500 mg/day (for an average sized person of 60 kg). Animal studies use metformin at doses up to 750 mg/Kg/day. Finally, in vitro cancer studies report using doses of metformin between 1 and 60 mM (for reviews, [91,92,93]). This has ignited a long debate on the relevance of “mitochondrial effects” in cancer experiments, with the idea that supra-pharmacological concentrations of metformin may “collapse” mitochondrial respiration, in an AMPK-independent and possibly, biologically irrelevant way. This debate has also stimulated the still ongoing search for a metformin mitochondrial transporter responsible for its accumulation into the inner membrane [92,94].

However, functional impairment of mitochondria was the most conserved feature observed when applying metabolomics to biopsies from advanced ovarian cancer patients treated with metformin, which were otherwise wildly heterogeneous [95], suggesting that mitochondrial targeting takes place and is relevant in vivo. Further, mitochondria-targeted analogues of metformin exerted clear anticancer effects [96,97,98]. In breast cancer cell lines that have undergone EMT, visual proof of mitochondrial targeting of metformin-analogues in fixed, intact cells has been obtained [99]. The copper- and iron-binding properties of metformin [29,100] were instrumental for its targeting to mitochondria and complex 1 dis-functioning [99]. Unfortunately these labeled metformin analogues were not used in parallel on untransformed cells, where previous biochemical fractionation suggested poor mitochondrial targeting of metformin [101,102]. Thus, there is still the possibility that the mitochondrial uptake of metformin is mechanistically different in untransformed cells and, possibly, influenced by the EMT status [103]. This would further support metformin’s therapeutic window. Finally, a reconciling piece of evidence is that, while supra-pharmacological doses (within the mM range) are required to induce antitumor effects in vitro, metformin intra-tumor concentrations in vivo were reported to be equal to those reached in plasma in orally-administered mice [104]. Thus, not surprisingly, tissues behave much differently from cells in vitro. Possibly, the glucose- and growth factor-rich conditions typical of in vitro cell culture media, and shown to dampen the effect of metformin, may cause the need for supra-pharmacological doses of the drug, in vitro [105]. After these considerations, we believe it is safe to recognize mitochondrial targeting as a first site of action for metformin in cancer cells. 

#### 3.2.2. Mitochondria Targeting Is Central to Metformin Anticancer Effects

Mitochondrial function is essential for tumor growth [106]. In fact, anabolic metabolism is crucial to support proliferation and survival [107] and mitochondria provide key fluxes of building blocks for macromolecule synthesis [108]. Additionally, mitochondria dynamically release key onco-metabolites, such as 2-hydroxybutarate (2HG), succinate, and fumarate, instrumental for the survival and networking of cancer cells in stress conditions [109,110]. Thus, mitochondria in cancer are a critical hub where stress-induced adaptive changes, including resistance to therapy, converge [111]. Besides functioning as an adaptive rheostat, these organelles are actively involved in supporting the survival of cancer cells through networking with the tumor microenvironment (TME) cell subpopulations, like cancer associated fibroblasts (CAFs) [112]. The way metformin alters the mitochondrial coupling at the electron transfer complex (ETC) is likely to be very similar to that discussed in non-cancer patients. In fact, the mitochondrial uncoupling effect of metformin triggers NAD+/NADH reduction and aspartate depletion [113], blocks the tricarboxylic acid cycle (TCA)-driven de novo fatty acid synthesis [114] and alters the adaptive response to hypoxia [115].

Still, AMPK activation by metformin with its opposite actions on mTORC1 and mTORC2 is the most studied mechanism of action of the drug [49,50]. On a molecular level, the “canonical pathway” consisting of LKB1-dependent and AMPK-dependent growth inhibition, consequent to the mitochondrial uncoupling effects of the drug, was the first mechanism to be considered in cancer [91]. Among the AMPK effectors, we consider, firstly, the effect of metformin-activated AMPK towards molecules and processes involved in stress adaptation; additionally, we consider metformin’s effect, first on bulk cell populations and, next, on specific cancer cell subpopulations. 

## 4. Metformin May Impair the Tumor Response to Stress

### 4.1. Metformin, p53, STAT3, NFkB, ER-Stress and Other Stress Adaptive Processses

#### 4.1.1. Metformin and p53

Collectively, wild-type p53 inhibits the shift from oxidative phosphorylation to glycolysis [116,117], promotes fatty acid oxidation while preventing lipid accumulation in cancer cells [118] and does so by modulating hundreds of targets [119]. For example, p53 upregulated expression of sestrin1/2 with consequent AMPK activation and inhibition of mTORC1 [120]. Further, gut specific activation of wtp53 revealed a transcriptional program enriched for OXPHOS functions [121]. On the other hand, loss of p53 elicited a Warburg-like metabolism [122,123]. Metformin-stimulated AMPK promoted p53 phosphorylation on Ser^15^ to trigger cell-cycle arrest [124]. Additionally, phosphorylation of human mouse double minute X (MDMX) on Ser^342^, leading to p53 stabilization, has been reported [125]. p53 stabilization was observed also in melanoma and lymphoma cells [126,127]. Stabilization and nuclear targeting of p53 by metformin may mediate, at least in part, the anti-proliferative and anti-glycolytic actions of the biguanide in cancer cells. What is less clear, is how the OXPHOS promoting activities of metformin-activated p53 match the mitochondrial dysfunction induced by metformin. Of note, is that the timing of the events (mitochondria uncoupling vs. transcriptional modulation) is different, but more investigation on this matter would be desirable. 

#### 4.1.2. Metformin and STAT3

Signal transducer and activator of transcription 3 (STAT3) is involved in many aspects of cancer adaptation to micro-environmental stress [128]. STAT3 shifts metabolism towards aerobic glycolysis via increased transcription of hypoxia inducible factor 1α (HIF-1α) [129]. Furthermore, the recently recognized mitochondrial targeting of STAT3 suggests that the action of the protein is even more complex and possibly phosphorylation dependent [130,131]. In fact, tyr^705^-STAT3 has been involved in mitochondrial gene transcription (OXPHOS genes), while the ser^727^-STAT3 was shown to modulate mitochondrial influx of calcium and to increase the efficiency of the ETC (for a review, [132]). Metformin, AMPK dependently, reduced both Ser^727^ and Tyr^705^ phosphorylation of STAT3 in four triple negative breast cancer (TNBC) cell lines [133]. Additionally, it reduced Tyr^705^ and/or Ser^727^phosphorylation in eight primary breast cancer cell cultures [134] and decreased Tyr^705^ and Ser^727^phosphorylation in glioblastoma, bladder cancer, cholangiocarcinoma and castration-resistant prostate cancer cell lines [134,135,136,137]. It would be interesting to assess whether metformin may interfere with mitochondrial OXPHOS at multiple levels, with early and late effects depending on the STAT3 phosphorylation status of the targeted cells.

#### 4.1.3. Metformin and NFkB

Metformin reduced the nuclear localization of nuclear factor kappa-light-chain-enhancer of activated B cells (NFkB)**,** with functional effects on proliferation and secretion of pro-inflammatory cytokines [134,138,139]. The relationship of NFkB with cancer metabolism is linked to mitochondrial [140] and p53 status. It ranges from increased glycolytic flux, to upregulation of OXPHOS in p53 deficient and p53 proficient contexts, respectively (for a review, [141]). In fact, impaired OXPHOS and mitochondrial function in primary chronic lymphocytic leukemia (CLL), and Richter syndrome cultures and mouse models treated with a preclinical grade NFkB inhibitor correlated with reduced nuclear accumulation of p65 [142]. A combined effect of metformin on p53 stability and NFkB localization may represent a further mechanism through which the drug alone, or in combination, would tackle the metabolic plasticity of cancer tissues. In line with this, as an effect of adaption to doxorubicin selection, MCF-7 cells reduced transcription of OXPHOS genes and increased nuclear localization of NFkB and p53. Encouragingly, metformin treatment reversed all these changes, and this correlated with restored sensitivity to the doxorubicin [84].

### 4.2. Metformin and Autophagy

Autophagy is a main cellular process which warrants cellular homeostasis in response to metabolic stress [143]. Defective or altered autophagy is clearly associated with disease states, including cancer. Interestingly, while increased autophagy exerts tumor suppressive functions at early stages of cancer development [144], in fully developed tumors it is largely employed for anabolic adaptive survival during therapy [145]. For instance, increased autophagy in the tumor microenvironment is increasingly regarded as relevant for tumor progression, as shown for colorectal and head and neck cancer cells [146,147]. Finally, chemo- and radio-resistant breast, ovarian, pancreatic and colorectal cancer cells were shown to upregulate autophagy under therapy-induced pressure [145]. Metformin induced autophagy, in gastric, liver cancer and myeloma cells through mTORC1 inhibition [148,149,150], or by inhibiting small mother against decapentaplegic-3 (SMAD3) phosphorylation in melanoma cells [151], at millimolar doses. AMPK phosphorylates forkhead boX O family of transcription factors-3 (FOXO3) at Ser413 or Ser588 and increases its nuclear localization and transcriptional activity [152] thereby contrasting the PI3K-AKT derived signals, which act oppositely and promote FOXO3 degradation [153]. FOXO3 modulates, transcriptionally and post-transcriptionally, autophagy-related genes (for a review, [154]). The effect of metformin-instigated AMPK on FOXO3 activation was observed in ovarian, breast and endometrial cancer cells [155,156,157]. 

The link between metformin and autophagy may be indirect and context specific; in fact, metformin was also shown, when used at micromolar doses and for a prolonged time on breast and cervical cancer cell lines, to cause autophagy inhibition by decreasing glutamine metabolism and ammonia accumulation [158]. While this discrepancy echoes what has already been discussed in this review, regarding the variety of dose-experimental settings adopted in cancer experiments, it is noteworthy that metformin seems to target a main Achilles’ heel of tumor cells: the glutamine addiction and its adaptive rerouting to sustain nucleotide synthesis (by providing alpha-ketoglutarate to the TCA cycle) and the glutathione (GSH) redox system [159,160,161]. Evidence for an effect of metformin on glutaminase (GLS) expression was collected in cervical cancer cell lines as well, possibly as an effect of c-MYC downregulation by the drug [162]. This is also interesting since the reduction of ammonia by metformin may also affect the tumor microenvironment [163].

### 4.3. Metformin and Mitophagy

Mitophagy is a specialized form of autophagy process supporting mitochondrial homeostasis. Damaged mitochondria exhibit reduced OXPHOS and increased ROS and are recognized by autophagy proteins and degraded in lysosomes (for a review, [164]). So, it is very likely that metformin effects the mitochondrial ETC and the increased ROS may trigger mitophagy. This may involve modulation of parkin-p53 [165,166]. Induction of mitophagy has been shown in diabetic patients treated with metformin [167]. Further, mito-metformin, a specifically mitochondria targeted metformin derivative [98], induced mitophagy features in CRC cells, in a k-RAS status-independent and AMPK-dependent way [97]. Interestingly, untransformed cells in the same experimental setting were much less affected [97].

### 4.4. Metformin and the Unfolded Protein Response (UPR)

Metformin treatment also impinges on the unfolded protein response (UPR) activity. This is a complex program whose activity increases in cells in response to the proteotoxic stress conditions emanating from the endoplasmic reticulum (ER), including glucose deprivation. The UPR response in cancer is linked to EMT, resistance and metastasis [168,169]. The cell response to UPR consists of reduced protein synthesis, increased protein degradation, induction of chaperone proteins and induction of pro-apoptotic effectors like C/EBP homologous protein (CHOP). Metformin, AMPK-dependently, blocked the activation of the UPR in acute lymphoblastic leukemia (ALL) cells and synergized with both AKT and the proviral insertion site in moloney murine leukemia virus (PIM-2) inhibitors [170].

### 4.5. Metformin and Modulation of microRNA Expression

A growing list of evidence supports a crucial role for non-coding RNAs and for their intracellular processing machinery in the metabolic reprogramming of primary or metastatic cancer cells [171,172]. Interestingly, microRNA modulation is an important part of metformin’s MoA: we and others have demonstrated that metformin controls a broad set of microRNAs by modulating the expression of the type III ribonuclease DICER [173], in an E2F-dependent manner [174]. Interestingly, microRNA modulation, which correlated with anti-cancer metabolic changes, was more prominent in breast cancer cell lines than in untransformed cells. This finding was validated in different experimental settings [175,176,177].

### 4.6. Attenuation of Stress Adaptation by Metformin Is Complex, Integrated and Tumor Context-Specific

Combined and converging targeting of these described adaptive mechanisms, in a tumor specific and to a tumor-specific extent, underlie the vast majority of reported effects of metformin on cancer cells. Some examples will follow. AMPK-mediated targeting of NFkB signaling was shown to mediate the growth-suppressive effect of metformin on hepatocellular carcinoma (HCC) development [178]. Additionally, the downregulation of acetyl-CoA carboxylase (ACC) and fatty acid synthase (FASN) were shown to contribute to the effects on HCC cells [56] and metformin was also shown to synergize with silencing of the hexokinase 2 (HK2) in a similar experimental system [179]. Further, metformin affected the chemo-resistance of cholangiocarcinoma cells by suppressing the cisplatin-ROS instigated increase in nuclear factor erythroid 2-related factor 2 (NRF2) [180] and inhibited proliferative and invasion potential through STAT3 and NFkB targeting [139]. Metformin induced esophageal squamous cell carcinoma (ESCC) cell autophagy and cell death via inhibiting the STAT3-B-cell lymphoma 2 (BCL2) pathway [181] and inhibited migration and invasion of ESCC cells by affecting NFκB nuclear localization [182]. In pancreatic cancer, the effect of the drug on the specificity protein-1(SP) transcription factors, and the effect on insulin receptor intracellular signaling were shown to be effective at inhibiting cancer growth. In a similar setting, metformin also synergized with aspirin to suppress BCL2 and myeloid cell leukemia 1 (MCL1) and augmented the effect of gemcitabine through inhibiting extracellular signal-regulated kinase (ERK)-p70S6K [183,184]. Metformin interference with the ERK-NFkB axis has been shown to attenuate the resistance of non-small cell lung cancer (NSCLC) cell lines to both gefitinib and a third-gen epidermal growth factor receptor (EGFR) tyrosine-kinase-inhibitors (TKI) [185,186]. Further, resistance of rectal cancer cell lines to both irradiation and 5-fluorouracile (5-FU) was attenuated by metformin via inhibition of STAT3 and transforming growth factor beta (TGF-β) [187] and colorectal cancer cells were sensitized to imatinib through metformin-mediated autophagy induction [188].

A common theme, besides metformin’s tumor-context specific actions, is the possibility to counteract stress-induced reprogramming. For instance, in NSCLC, cisplatin treatment was shown to induce ROS-mediated metabolic reprogramming, occurring early after treatment and this observation was confirmed in a few post-treatment patient biopsies and in two patient-derived-xenograft (PDX) models. This consisted of increased OXPHOS and augmented mitochondrial mass with increased levels of peroxisome proliferator-activated receptor gamma coactivator 1-alpha (PGC1α) [189]. This chemotherapy-instigated OXPHOS switch was also observed in colon cancer models [190,191] and in ovarian cancer models [192]. Additionally, the early adaptive process of acquired resistance of the EGFR-mutant NSCLC to tyrosine-kinase-inhibitors (TKI), was linked to increased mitochondrial mass and performance of OXPHOS [193]. Intriguingly, in the case of NSCLC, it was shown that metformin treatment could effectively counteract such an adaptive OXPHOS switch, with chemo-sensitizing effects in vitro and in vivo, to cisplatin [189].

## 5. Metformin and Cancer Stem Cells: Another Layer of Complexity

The term cancer stem cells (CSCs) refers to specific subpopulations within a tumor, endowed with greater plasticity and adaptive potential (as compared to the majority of the cells composing the bulk tumor cell population) [194]. CSCs are considered responsible for resistance to therapy and, therefore, relapse of the tumor. Therapy-induced stress provides a selective pressure, fueling survival and the emergence of CSCs through complex paracrine interactions with the TME [195,196,197]. The maintenance of a stable NADH/NAD+ ratio is instrumental for the adaptive properties of CSCs, and takes place through modulation of the glycolytic, OXPHOS and TCA cycle. Notably, putative CSC subpopulations have been metabolically categorized as either mainly glycolytic or mainly dependent on OXPHOS (for a review, [12]). The reason for this, besides obvious technical and biological differences among the experimental systems adopted, may lie in the fact that there are no single cancer stem cells, but heterogeneous cell populations endowed with “stemness” traits; their plasticity and capacity of dynamic interactions with the TME may dictate their fluctuating metabolic behavior, which may therefore become tumor-stage and -time specific (Lee et al., 2015). Pertinent to this, the cancer stem cell concept [197] inspires the potential usefulness of exploiting combinatorial approaches, which have recently been shown to provide interesting results. Thus, the combination of metformin with 2-deoxy-d-glucose (2-DG), a glycolysis inhibitor, or the use of intermittent fasting as a strategy to reduce glucose were shown to synergize with metformin treatment in vitro and in vivo, in breast cancer models [198,199,200]. Other synergic action towards the CSC-linked metabolic heterogeneity were observed with caffeic acid (trans-3,4-dihydroxycinnamic acid, CA) and dichloroacetate (DCA). In cervical cancer cell lines, CA was shown to synergize with metformin at inhibiting de novo fatty acid synthesis thereby decreasing the amount of unsaturated long chain fatty acids [201]. CA and metformin were shown to attenuate TGF-β1-induced EMT in cervical carcinoma cells [202], partially because of the involvement of unsaturated fatty acids to support EMT and the emergence of CSC [203,204]. DCA was shown to potentiate the effect of metformin in breast, ovarian cancer and glioma cells in vitro and in vivo [205,206,207]. Further, both DCA and CA synergized with metformin [115,162,205] to activate the pyruvate dehydrogenase complex (PDH) by inhibiting pyruvate dependent kinase (PDK), thereby promoting the oxidative decarboxylation of pyruvate to acetyl-CoA and enhancing the mitochondria-generated ROS. This counteracts the HIF-1α-dependent induction of PDK in favor of glycolytic processes in the hypoxic regions of the tumor [208]. Additionally, activation of PDH has been shown to reduce the CSCs in mouse mammary tumor virus (MMTV)-*Wnt*-1 mouse models [209].

### Metformin Attenuates Cancer Stem Cell Features

One compelling feature of the cancer stem cells is their ability to sustain therapy-induced stress: therefore, CSCs have been defined as chemo-resistant or radio-resistant, according to the experimental system used. Cancer stem cells survive chemotherapy in vivo. In ovarian cancer patient samples, a transient enrichment for ALDH1A1, CD44 and CD133^pos^ cells was recorded, after chemotherapy [210], and this was validated by other studies in treated cancer patients [211]. Thus, interference with this property may result in collective chemo- and radio-sensitization: generally, this is accompanied by disappearance “or reduced expression” of surface markers employed for identifying the CSCs, which may indicate eradication of the positive clones. This is the case, for example, for CD44^+^/CD24^−^ cells in breast cancer cells [84,105,212], for CD133+ CSCs in pancreatic CSCs [213] and for glioblastoma CD133+ CSCs [214]. Cell subpopulations endowed with high aldehyde dehydrogenase (ALDH) activity have been shown to be chemo-resistant and to possess some stemness traits, in many tumors including sarcoma, lung and breast [215]. Interestingly, purified ALDH^high^ (or “bright”—based on fluorescence levels) cells were shown to have higher mitochondrial mass, both in vitro and in vivo [216]. We treated FACS-sorted ALDH^bright^ cells from breast cancer cell lines with metformin and found that, in fact, metformin elicited substantially different metabolic changes, as compared to the bulk population [217]. Notably, metformin action pushed the ALDH^bright^ cells towards a more glycolytic phenotype (possibly to compensate for mitochondrial inhibition), more similar to what was observed in the more differentiated, chemo-sensitive ALDH^low^ cells [217], in agreement with the larger mitochondrial mass [216] and metabolic plasticity of CSCs [218,219].

A selective action of metformin towards CSC cell subpopulations has been shown in the past, in a clear way at least in breast cancer, ovarian, prostate and lung cancer models [212,220,221,222]. What confers a relative selectivity of action towards CSC cell subpopulations (as compared to non-CSC from the same culture or animal or patient) is an interesting question. The molecular determinants for the specificity of metformin towards those cell subpopulations are still to be determined. One observation is that, generally, such effects are not “black or white” and consist rather of a higher sensitivity of CSCs than no sensitivity of the non-CSC cell subpopulations, to metformin treatment. It is likely that metformin’s specificity is driven by the different biological status of the CSCs. For example, in pancreatic cancer stem cells (CSCs), specificity of action seems to be conferred to the drug by their distinct metabolic phenotype. CSCs were indeed shown to be mainly endowed with oxidative metabolism (OXPHOS) due to elevated levels of PGC-1α consequent to c-MYC suppression (as compared to the mainly glycolytic non-CSC cell subpopulations). Treatment with metformin caused mitochondrial inhibition and apoptosis, specifically in those CSCs. Metformin resistant clones arise during treatment and exhibited downregulated levels of PGC-1α and, intriguingly, a metabolic phenotype sitting between a glycolytic and an OXPHOS one, again supporting the great plasticity of these cells [223]. Given the tight relationships between epithelial to mesenchymal transition and the generation of CSCs [224], and the high sensitivity of EMT driven or mesenchymal breast cancer cell lines to metformin [84], one possibility is that cells undergoing such a program may expose metabolic targets to metformin. Metformin treatment strongly reduced the teratogenicity of mouse embryo fibroblast-derived IPSs without affecting the multipotency of those cells. This was due to the effect of metformin on OCT4-driven signals, responsible for malignant transformation [225]. OCT-4 dependent signals are considered as important determinants of the CSC phenotype (for a review, [226]).

In line with this, a recently closed phase II clinical trial has evaluated the impact of metformin on ALDH-CD133^pos^ CSC number and on carcinoma-associated mesenchymal stem cells (CA-MSCs) in 38 non-diabetic patients with advanced-stage epithelial ovarian cancer (EOC) [227]. The study revealed that the tumors from patients treated with neoadjuvant metformin, with or without chemotherapy, before surgery, exhibited a more than two-fold reduction in ALDH-CD133^pos^ ovarian CSCs [227]. Despite this, as generally happens for phase II studies, this study did not have a control arm and had a small sample number, the overall survival of the metformin-treated patients was better than expected, based on historical controls [227]. Last but not least, the authors observed an interesting effect on the methylation status of the cancer-associated-mesenchymal-stem-cells (CA-MSCs), known to provide essential pro-tumorigenic signals to ovarian cancer cells. As somehow expected, the authors encountered a significant inter-tumor heterogeneity which prevented statistical significance. However, they found that CA-MSCs from a subgroup of metformin treated tumors were capable of chemo-sensitizing, in vitro, a co-cultured epithelial ovarian cancer cell line (as compared to non-metformin treated CA-MSCs and metformin unresponsive CA-MSCs) [227]. The effect of metformin on the ovarian cancer CA-MSCs methylation profile opens up another relevant point: that metformin may modify the TME-promoting activity towards CSCs. Evidence in favor of such effects are emerging [228,229]. Metabolic action on CA-MSCs and other TME cell populations may alter the respiratory fitness of TME cell populations and their multifaceted pro-tumorigenic actions [230], thereby attenuating the metabolic symbiosis between TME components and CSCs [231]. Additionally, the effect of metformin on microRNAs could alter, for example, the exosome composition which has been shown as an important part of the TME cancer interaction [232].

Last but not least, CSCs are characterized by elevated levels of autophagy when compared to more differentiated cancer cell populations, an observation confirmed in multiple cancer types [233] and exacerbated by therapy-induced stress [234,235]. Whether metformin modulates autophagy and mitophagy differently in cancer stem cells as opposed to more differentiated cancer cells, would be an interesting question to answer. This would be a further way metformin may interfere with the extreme plasticity of these cell subpopulations.

## 6. Concluding Remarks

In summary, metformin shows a complex and partially unexplored MoA. Targeting of mitochondria, interference with redox systems and cytosolic signaling all converge into a suggestive anticancer effect, which starts from metabolic actions and impinges on developmental signaling (EMT) and tumor–stroma crosstalk. However, the need for combinatorial therapies and the non-homogeneous results of the clinical trials, all prompt towards the need for further dissecting its action in cancer patients in light of tumor heterogeneity. If metformin is capable of targeting specific cell subpopulations within the tumor, thereby dampening their adaptive properties, then the search for a biomarker to stratify patients based on the degree of representation of such cell subpopulations may be the next welcome step. Obtaining specific metabolic and pharmaco-genomic signatures would pave the way for this search. Very recently, specific methylation signatures associated with metformin response and tolerance, in naïve, type 2 diabetic patients, from blood and from adipose tissue have been documented [236]. Additionally, as mentioned earlier, specific genomic signatures of resistance/sensitivity were obtained from a limited number of cancer cell lines [86]. This leaves hope that stratification of cancer patients based on their responsiveness to metformin may be possible in the near future.
